# Case report on an extremely rare type of ependymoma arising from the thigh

**DOI:** 10.1016/j.ijscr.2024.110301

**Published:** 2024-09-15

**Authors:** T. Furuta, T. Sakuda, K. Yoshioka, A. Oda, A. Ishikawa, N. Adachi

**Affiliations:** a Department of Orthopaedic Surgery, Hiroshima University, Graduate School of Biomedical and Health Sciences, Hiroshima, Japan; b Department of Molecular Pathology Institute, Hospital: Graduate School of Biomedical and Health Sciences, Hiroshima University

**Keywords:** Ependymomas, Thigh, Surgery, Adjuvant radiotherapy, Extra-neural Ependymomas

## Abstract

**Introduction:**

Ependymomas are neuroepithelial neoplasms of the central nervous system that arise from the precursor cells lining the ventricular system and the central canal of the spinal cord. Herein, we report a case of an extremely rare type of ependymoma arising from the thigh. Then, a literature review was performed.

**Presentation of case:**

An 87-year-old female Japanese patient presented with a chief complaint of a mass on the medial aspect of her right thigh. Pathology revealed a grade 2 extra-neural ependymoma. PET-CT and brain MRI showed no neoplastic lesions in the central nervous system, and the tumor was localized only in the right thigh. The tumor was growing and was treated as a low-grade tumor with extensive resection and postoperative adjuvant radiotherapy. The patient has been alive for 3 years, without postoperative recurrence or complications.

**Discussion:**

This case presents a rare extra-neural ependymoma of central nervous system origin arising in the thigh. The pathogenesis is unknown, but a search for neoplastic lesions in the cerebrospinal cord is warranted. Extra-neural ependymomas should be treated as low-grade tumors because they are more prone to recurrence and metastasis than ordinary ependymomas.

**Conclusion:**

We experienced an extremely rare extra-neural ependymoma arising in the thigh. A search for tumors in the central nervous system region of the cerebrospinal cord, previous literature, and clinical, imaging, and pathological findings should be consulted to determine a treatment strategy.

## Highlights

• Ependymomas are neuroepithelial neoplasms of the central nervous system.
• Experienced extra-neural ependymoma arising from the thigh that has very rarely reported.
• Ependymomas located other than above or below the tent are considered Grade II.
• The patient was treated with low grade malignant tumor(gradeIII) based on clinical findings of expansion tendency.

## Abbreviations

MRI: Magnetic resonance imaging
PET-CT: Positron emission tomography-computed tomography

## 1 Introduction

Ependymomas are neuroepithelial neoplasms of the central nervous system that arise from the precursor cells lining the ventricular system and spinal cord central canal. Overall, ependymomas account for 2 %–3 % of central nervous system tumors, 8 %–10 % of pediatric central nervous system tumors, and < 4 % of adult central nervous system tumors [1,2]. These tumors are classified into grade I (subependymomas and myxopapillary ependymomas), grade II (classic ependymomas), and grade III (anaplastic ependymomas) according to the degree of atypia (degree of morphological abnormality) of tumor cells [3].

In general, grade II or III ependymoma is found in the cerebrum above the tent and in the posterior fossa below the tent, with tumors being more frequently observed in the lower tent. Lesions arising from the spinal cord are classified as grade II [3]. There is no standard staging system for ependymoma. It is classified according to the WHO 2021 system, which takes into account histopathologic and molecular features. They are classified as supratentorial, subintentorial, and spinal ependymomas. Supratentorial ependymomas are classified based on specific fusion types such as ZFTA (formerly RELA) and YAP1, while subintentorial ependymomas are classified into type A and B based on methylation patterns, with type A being more common in adolescents and young adults and having a worse prognosis. In a small percentage of spinal ependymomas, MYCN amplification is considered to be a prognostically worse factor that reduces survival [4,5]. Pathologically, ependymomas exhibit a ventricular ependymal cell morphology, and they are characterized by an epithelial-like arrangement of columnar cells. The characteristic cellular arrangements include ependymal rosettes and perivascular pseudorosettes. Ependymomas test positive for glial fibrillary acidic protein (GFAP), epithelial membrane antigen (EMA), S-100, and vimentin on immunohistochemistry [6,7]. However, regardless of tumor grade and location and patient age, all patients should be treated with maximal surgical resection and adjuvant radiotherapy. Approximately 20 %–30 % of cases are challenging to manage due to recurrence or metastasis. Herein, we report a primary extra-neural ependymoma on the thigh without central nervous system cells, Extra-neural ependymomas have been reported to occur in the pelvis, sacrum, and groin, but not in the thigh [[8], [9], [10]]. the treatment of this extremely rare extra-neuralependymoma was discussed, and a literature review was performed. The patient provided informed consent for the publication of this article. Treatment interventions were performed in compliance with SCARE 2023 [11].

## 2 Presentation of case

An 87-year-old female Japanese patient visited a nearby hospital after she found a mass on the medial side of her right thigh several months back. She had no significant family and medical history.

**Fig. 1:**
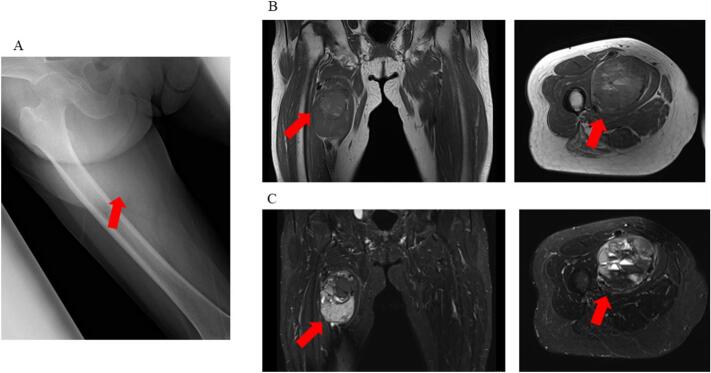
X-ray and preoperative magnetic resonance imaging of ependymoma in the proximal medial thigh. (A) A swollen soft shadow was seen on the medial side of the right thigh. (B) T1-weighted images show a low signal throughout the interior of the tumor (arrowhead). (C) T2-weighted images show a mixture of low- and high-signal areas inside the tumor, and multiple niveau formations were also observed (arrowhead).

**Fig. 2:**
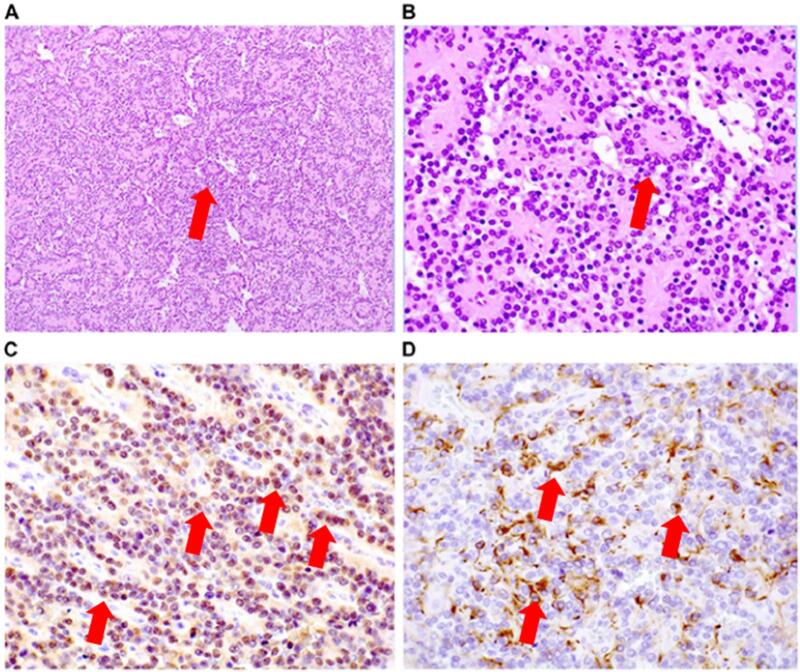
Pathological findings of ependymoma in the thigh. (A, B) Hematoxylin and eosin staining. Original magnification: (A) 100×, (B) 400×. (C, D) Immunohistochemical staining. (C) S-100, (D) GFAP, original magnification: 400 × .

**Fig. 3:**
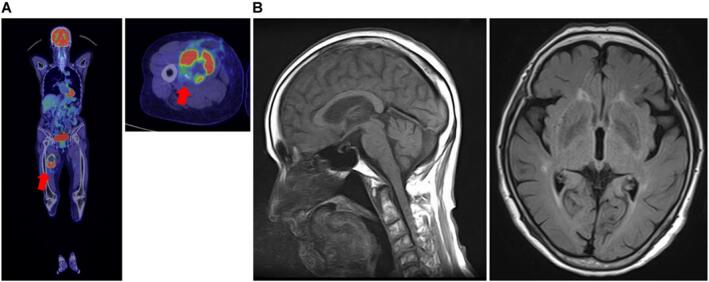
Preoperative general examination. (A) Positron emission tomography-computed tomography scan did not show abnormal accumulation except in the right thigh (SUVmax: 14.1). (B) T2-weighted FLAIR image of brain MRI showed no neoplastic lesions.The only neoplastic lesion was in the right thigh, and there was no tumor in the central nervous system region.

The patient underwent X-ray and magnetic resonance imaging (MRI) based on the discretion of her previous physician. Results showed a 7-cm tumor adjacent to the femoral artery, femoral vein, and nerve (Fig. 1), with low T1 and T2 signals and a high signal on the medial right thigh. Next, incisional biopsy at the previous hospital was performed. Based on the pathological results, the patient was diagnosed with a glomus tumor. Further, she was referred to the Department of Bone and Soft-Tissue Tumor Surgery because the clinical and imaging findings were inconsistent with the pathological findings. An extremely firm, immobile tumor was found on the medial side of the right thigh. The patient did not present with tenderness or burning. Clinically, The tumor was growing rapidly. Upon examination, the lesion size was 7 cm (>5 cm). Thus, soft tissue sarcoma, desmoid, Soft tissue metastasis, malignant lymphoma, Giant cell tumor, hemangioma etc. was suspected, and another incisional biopsy was performed. Pathological examination revealed an epithelial-like arrangement of columnar cells, characterized by ependymal rosettes and perivascular pseudorosettes, as shown in Fig. 2A and B. Immunohistochemically, the cells tested positive for GFAP,S-100 as shown in Fig. 2C and D (Fig. 2). This was determined to be an extra-neural ependymoma. Computed tomography (CT) scan and positron emission tomography (PET)-CT scan were performed, and no other lesions were found. Head MRI also confirmed the absence of neoplastic lesions (Fig. 3). After consultation with the clinical oncology and pathology departments, the tumor was diagnosed as grade II ependymoma. For gradeII, a marginal resection would appropriate. However, the clinical findings showed a trend toward tumor enlargement and neurovascular invasion. Based on previous literature, it was concluded that it was reasonable to consider the extra-neural ependymoma to be a grade III, or low-grade malignant tumor [[8], [9], [10]]. We planned the most extensive resection possible and adjuvant radiation therapy based on the intraoperative gross findings and postoperative pathology. Surgery was performed with neurovascular preservation and as extensive resection as possible. Because of the thin adductor muscle and vastus medialis muscle present between the tumor and the skin, a skin flap was not planned.

**Fig. 4:**
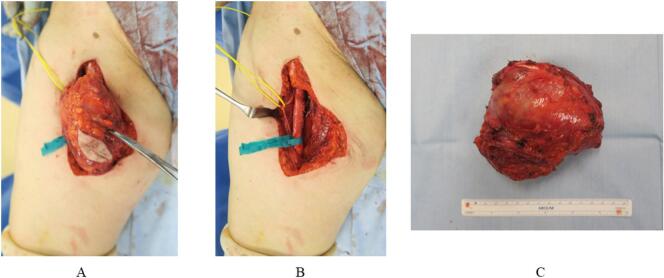
Intraoperative images. (A) Image taken just prior to the removal of the tumor from the medial side of the right thigh. (B) Image taken after the tumor was resected, with blue tape covering the femoral arteriovenous vein and yellow tape covering the femoral nerve. (C) Resected tumor removal, without a ruptured tumor membrane. (For interpretation of the references to colour in this figure legend, the reader is referred to the web version of this article.)

The approach was to make a longitudinal skin incision just above the tumor on the medial thigh and resect the thinned vastus medialis and adductor muscles with the tumor side. Deeply, the neurovascular bundle was very adherent, and the tumor was resected with the extravascular membrane and nerve sheath adhering to the tumor capsule attached to the tumor side. (Fig. 4). Tumor margins were negative. However, because of the strong adhesion grossly, the patient was managed with radiotherapy, which was administered in 30 sessions at a dose of 2 Gy with a total of 60 Gy.

**Fig. 5:**
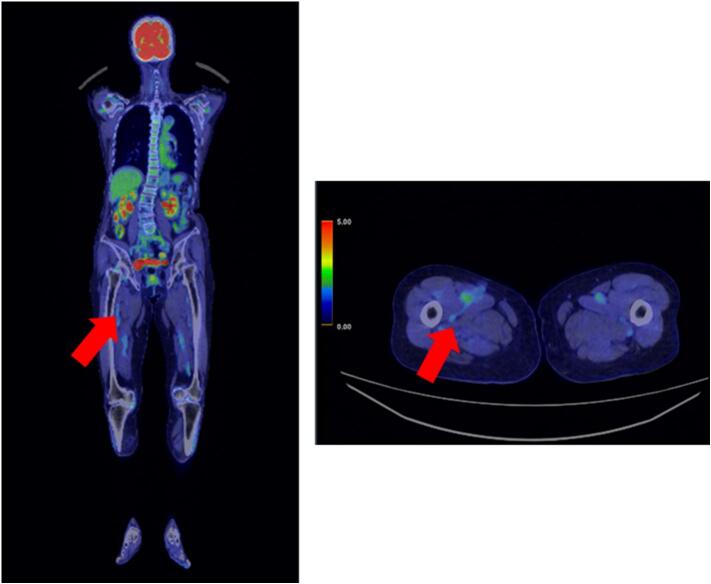
PET-CT scan 3 years postoperatively, PET-CT showed no detectable tumor lesion on the medial side of the right thigh (arrow) and no metastasis.

Three years after treatment, a PET-CT scan was performed and no recurrence or metastasis was found in the patient (Fig. 5) Nevertheless, she is still undergoing cautious follow-up.

## 3 Discussion

Ependymomas are neuroepithelial neoplasms of the central nervous system that arise from the precursor cells lining the ventricular system and spinal cord central canal. In general, a grade III lesion is more malignant and has a poorer prognosis than the gradeIorII types [[1], [2], [3]]. In other words, it thinks in a low-grade malignant tumor. However, in the current case, the pathology was grade 2, but the tumor tended to increase in size and was considered to be grade 3 based on the clinical findings. Furthermore, the ependymoma in this case occurred on the thigh, where there are no central nervous system cells, and extra-neural ependymomas have been reported very rarely [[8], [9], [10]].The first and most important question is whether the pathology is truly consistent with ependymoma. Pathological examination revealed an epithelial-like arrangement of columnar cells, characterized by ependymal rosettes and perivascular pseudorosettes. Immunohistochemically, the cells tested positive for GFAP, S-100, EMA and vimentin. The tumor was located in the thigh. However, it tested positive for GFAP, an astrocytic marker in the brain, and S-100, a neuronal marker. Therefore, it is a central nervous system tumor. These findings are consistent with those of extra-neural ependymomas. ZFTA (formerly RELA) and YAP1, methylation patterns, and MYCN searches may have provided clues to predict the origin of the extra-neural ependymoma [5]. In other words, it may be possible to infer whether supratentorial, posterior fossa (PF), or spinal cord metastasized to the femur, etc. However, it was not performed because of cost issues and because it would not affect the treatment strategy for the femoral region [[5], [6], [7]].

Second, there is no standard staging system for ependymomas, although staging is thought to be the process of determining how a cancer has spread. It is classified according to the WHO 2021 system, which takes into account histopathologic and molecular features. They are classified into ependymal, supratentorial, posterior fossa (PF), and spinal cord tumors. It was an extra-neural ependymoma that did not belong to any classification. Although rarely reported, it was recommended that extra-neural ependymomas be treated as low malignant grade [[8], [9], [10]]. we decided to treat the patient as a grade III or low-grade malignant tumor and to perform as wide a resection as possible and to add adjuvant radiotherapy according to the intraoperative gross findings and postoperative pathological findings. After a wide resection as possible, although the pathology was negative for margins, the patient underwent adjuvant radiotherapy because of a strong tendency toward adhesion around the vascular nerve bundle. There were no postoperative recurrent metastases and no postoperative complications. We believe that the treatment plan was appropriate [[12], [13], [14], [15]].

Third, it is important to consider whether and how the tumor was located in the thigh, where there are no central nerve system cells, and this is what prompted this report. In the literature, there are several reports on malignant tumors with residual metastases and spontaneous regression of the primary tumor. Malignant tumors that have spontaneously regressed include testicular germ cell tumor, renal cell carcinoma, melanoma, basal cell carcinoma, neuroblastoma, and colon and breast tumors. Spontaneous regression of malignancy occurred in approximately 1 in 6000–10,000 cases. The actual cause of spontaneous tumor regression is not completely understood. However, tumor degeneration produces immunity, which may contribute to spontaneous regression, or severe infection may cause sagging and necrosis of the carcinoma, leading to spontaneous regression [[16], [17], [18], [19], [20], [21]]. Ependymomas have also been reported to metastasize to the lungs, thighs, and ribs in this case, we considered the possibility that the primary tumor originated from the brain or spinal cord, metastasized to the thigh, and then regressed to the primary tumor. To rule out the possibility of missing the primary tumor, PET-CT and brain MRI scans were performed. Results confirmed the absence of a tumor in the central nervous system of the brain or spinal cord.

This case was a pathologically grade II, extremely rare extra-neural ependymoma arising from the thigh. It was important to discuss the case with the clinical oncologist and pathologist, to search for the pathogenesis of the ependmoma by brain and spinal cord imaging studies, to find an increasing trend in clinical findings, and to develop a treatment strategy based on the literature on extra-neural ependymomas.

## 4 Conclusion

We experienced an extremely rare extra-neural ependymoma arising in the thigh. Since ependymoma is of central nervous system origin, the treatment plan should be decided based on a comprehensive judgment including consultation with a clinical oncologist and pathologist to search for the cause of why the ependymoma occurred on the thigh, search for tumors in the central nervous system region of the brain and spinal cord, literature review of extra-neural ependymomas, clinical and imaging findings, and other factors.

## Ethical approval

This study is a case report, has been fully explained to and consented by the patient, and was determined not to require ethical approval under the ethical approval guidelines of Hiroshima University Hospital.

## Funding

No external funding was received for the publication of this paper.

## Author contribution

Taisuke Furuta will contribute to the research concept and design, data collection, analysis and interpretation, oversight and leadership responsibility for planning and execution of research activities, and external mentoring of the core team. We would like to thank the following for their contributions to this work: Enago (www.enago.jp), for English language review. Tomohiko Sakuda contributed to the data collection of the study. Koki Yoshioka contributed to the data collection, research concept, and treatment design of the study.

Akira Ishikawa contributed to pathological diagnosis and data collection.

Nobuo Adachi contributed to the concept, supervision, and guidance of the study.

## Guarantor

Tomohiko Sakuda

TEL: +81-82-257-5233, FAX: +81-82-257-5234

E-mail: sakuuda@yahoo.co.jp

Koki Yoshioka

TEL: +81-82-257-5233, FAX: +81-82-257-5234

E-mail: rororosso.nero92@gmail.com

Taisuke Furuta

TEL: +81-82-257-5233, FAX: +81-82-257-5234

E-mail: fu09100913@yahoo.co.jp

## Research registration number

Not required for this report.

## Declaration of competing interest

The authors declare no conflicts of interest.
